# Integrin α10 regulates adhesion, migration, and osteogenic differentiation of alveolar bone marrow mesenchymal stem cells in type 2 diabetic patients who underwent dental implant surgery

**DOI:** 10.1080/21655979.2022.2079254

**Published:** 2022-05-29

**Authors:** Chao Liang, Xiu Liu, Changying Liu, Yifan Xu, Wei Geng, Jun Li

**Affiliations:** a Department of Dental Implant Center, Beijing Stomatological Hospital, School of Stomatology, Capital Medical University, Beijing, China; b Beijing Institute of Dental Research, Beijing Stomatological Hospital, School of Stomatology, Capital Medical University, Beijing, China

**Keywords:** Dental implant, type 2 diabetes mellitus, bone marrow mesenchymal stem cells, ITGA10, osteogenic differentiation, focal adhesion pathway

## Abstract

Type 2 diabetes mellitus (T2DM) is a clinically important risk factor for dental implant treatment failure. An imbalance in the microenvironment of the jawbone of diabetic patients can impair the functions of bone marrow mesenchymal stem cells (BMSCs), thereby interfering with implant osseointegration during the healing phase. This study aims to investigate potential molecular factors associated with abnormal BMSC biological functions in diabetic patients with dental implant failure and identify intervention targets to improve implant osseointegration. The results of cell adhesion, cell scratch migration, alkaline phosphatase (ALP) activity, alizarin red staining, and real-time PCR assays showed that the adhesion, migration, and osteogenic differentiation abilities were significantly lower in alveolar BMSCs isolated from diabetic patients with implant failure (DM-F group) than in those isolated from diabetic patients with implant success (DM-S group) and normal patients (Nor group). Also, bioinformatics analysis and verification of whole-cell proteomics results revealed that integrin subunit alpha10 (ITGA10) expression in BMSCs was significantly lower in the DM-F group than in the DM-S group and much lower than that in the Nor group. In addition, lentiviral mediated short hairpin RNA (shRNA) or complementary DNA (cDNA) was used to knockdown or overexpress *ITGA10* in BMSCs from diabetic patients, and the results revealed that *ITGA10* knockdown significantly reduced the adhesion and migration abilities of BMSCs and inhibited their osteogenic differentiation potential by disturbing the FAK/PI3K/AKT/GSK3β/β-catenin pathway. *ITGA10* overexpression produced the opposite results. In summary, this study revealed that low ITGA10 expression in BMSCs from the DM-F group is a major cause of cell dysfunction, including reduction in the adhesion, migration, and osteogenic differentiation abilities of BMSCs, and provided insight into the underlying mechanism. Additionally, ITGA10 was identified as a potential target protein for improving the biological functions of BMSCs and dental implant osseointegration in T2DM patients.

## Graphical abstract

**Figure:**
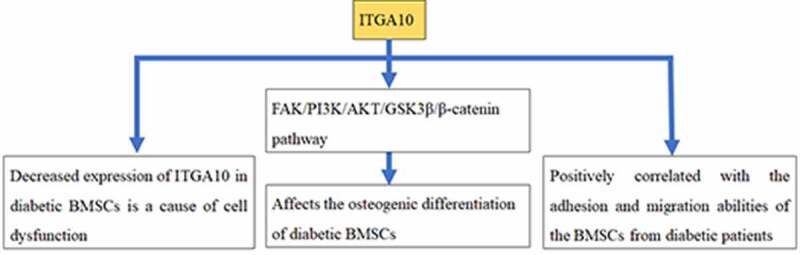

## Highlights

- A decreased expression of ITGA10 in diabetic BMSCs is a cause of cell dysfunction
- ITGA10 was positively correlated with the adhesion and migration abilities of BMSCs
- ITGA10 affected the osteogenic differentiation of BMSCs by the focal adhesion pathway

## 1. Introduction

With the development of oral implantology, dental implant restoration has become the preferred method for restoring oral function in patients with dental defects or edentulous patients [1]. However, type 2 diabetes mellitus (T2DM) has been identified as an important risk factor affecting the survival rate of dental implants [2–5]. Patients with diabetes are often affected by different degrees of bone tissue remodeling disorders, which interfere with implant osseointegration during the healing stage, resulting in a significantly higher failure rate among diabetic patients than among nondiabetic patients [6]. Long-term clinical studies have also found that the 20-year implant retention rate of T2DM patients is approximately 70%, which is far lower than that (more than 90%) of nondiabetic patients [7]. To date, the negative effect of diabetes mellitus on implant osseointegration and long-term retention is still a challenge for dentists and their patients [8].

Bone marrow mesenchymal stem cells (BMSCs) are adult stem cells that play important roles in implant osseointegration processes [9,10]. Studies have shown that a high-glucose environment has a substantial negative impact on BMSC biological processes, such as cell proliferation and migration [11], and that glucose dose-dependently inhibits the osteogenic differentiation potential of BMSCs [12,13]. However, microenvironmental changes in the jawbone of T2DM patients are complex [14]. The cause of dental implant osseointegration failure in T2DM patients is certainly not high glucose alone, and the molecular mechanisms of BMSC dysfunction and reasons of high implant failure rate in T2DM patients during the healing stage still need clarification.

Our preliminary study investigated the causes of dental implant failure in alveolar BMSCs from bone particle specimens obtained during dental implant surgery in three groups of patients, including diabetic patients with implant failure (DM-F group), diabetic patients with implant success (DM-S group), and normal patients without diabetes (Nor group), which were cultured and analyzed by whole-cell proteomic analysis (data have been deposited in ProteomeXchange with the PXD016489 identifier) [15]. Among the identified differentially expressed proteins in this dataset, integrin subunit alpha10 (ITGA10) expression was significantly lower in the DM-F group than in the other two groups. Integrins are an important class of transmembrane proteins whose major function is to regulate cell adhesion through the recognition and binding of specific extracellular matrix (ECM) proteins [16]. The adhesion of BMSCs on the dental implant surface is the initial step of implant osseointegration, and regulating the expression of integrins in the cell membrane of BMSCs could be an effective strategy to improve cell adhesion and early implant osseointegration [17]. ITGA10 is a type II collagen-binding integrin that was first isolated from chondrocytes [18], and several studies have shown that ITGA10 expression in growth plates plays an important role in bone development [19–21]. However, to our knowledge, the effect of ITGA10 expression on the function of BMSCs has not been reported to date. Whether abnormal ITGA10 expression in alveolar BMSCs is an important cause of cell dysfunction and poor implant osseointegration in T2DM patients remains to be elucidated.

This study aimed to determine first the differences among patients in the DM-F, DM-S, and Nor groups, in biological processes in alveolar BMSCs, including adhesion, migration, and osteogenic differentiation, and then the molecular cause of abnormal BMSC functions in diabetic patients with implant failure. Subsequently, the effect of the identified differentially expressed protein ITGA10 on these processes in alveolar BMSCs from T2DM patients was evaluated, and the associated pathways were analyzed to determine the mechanism by which ITGA10 interferes with the osteogenic differentiation potential of alveolar BMSCs. Based on the findings of the above research, our study is expected to identify ITGA10 as a molecular intervention target for the improvement of BMSC functions and implant osseointegration in T2DM patients.

## 2. Materials and methods

### 2.1. Cell culture

The sources of human alveolar BMSCs from diabetic patients with implant failure (DM-F group), diabetic patients with implant success (DM-S group), and normal patients without diabetes (Nor group) were the same as those in the preliminary proteomic study [15]. There were five patients in each group; each diabetic patient with implant failure was strictly controlled for variables matched to a diabetic patient with implant success and a non-diabetic patient. Patients were all male, aged between 50 and 60 years old (age difference ±1 year), nonsmokers, and had no diagnosis of systemic diseases such as hypertension or hyperlipidemia. The glycosylated hemoglobin of patients in both the DM-F and DM-S groups was between 6% and 8% (diabetic patients need to maintain their glycosylated hemoglobin to less than 8% before undergoing implant surgery), enabling the experimental results to be unaffected by blood glucose. In addition, according to the currently acknowledged criteria for implant success [22, 23], a failed implant is defined as an implant with clinical mobility and peri-implant radiolucent imaging. In this study, the patients in the DM-F group developed these clinical manifestations within 3 months after the implant surgery and experienced implant dislodgement. Cells were isolated from otherwise discarded bone particle specimens obtained using a low-speed drilling (50 rpm, without irrigation) technique [24] during dental implant surgery. The study was reviewed and approved by the Ethics Committee of Beijing Stomatological Hospital, Capital Medical University (approval code: CMUSH-IRB-KJ-PJ-2018-08) and conformed to the Declaration of Helsinki. All patients provided written informed consent to participate in this study. The cells in the collected bone particles were cultured in 5 mL of mesenchymal stem cell medium (MSCM) (ScienCell Research Laboratories Inc., Carlsbad, CA, USA) in a 60-mm Petri dish (Corning Inc., NY, USA) incubated in a 37°C incubator with 5% CO2 for 7 days, and then, the medium was replaced every 3 days. In this study, third-passage alveolar BMSCs from the DM-F group, DM-S group, and Nor group were thawed and cultured for subsequent experiments.

### 2.2. Immunofluorescence staining

BMSCs were seeded in a 24-well plate with adhesive slides at a density of 3 × 10^4^ cells/well. After adhesion for 2 h, the cells were fixed with 4% paraformaldehyde, treated with 0.2% Triton (Sigma-Aldrich, St. Louis, MO, USA), blocked with 10% goat serum (ZSGB-BIO, Beijing, China), and incubated with diluted rabbit monoclonal anti-F-actin primary antibody (Abcam, Cambridge, UK) at 4°C, overnight. The cells were then incubated with rhodamine-conjugated goat anti-rabbit fluorescent secondary antibody (1:200; ABclonal, Wuhan, China). Cell nuclei were stained with 4’,6-diamidino-2-phenylindole (DAPI; ABclonal). The images were acquired using a fluorescence microscope (Olympus Corporation, Tokyo, Japan) at 200× magnification.

### 2.3. Cell adhesion assay

BMSCs were seeded in 6-well plates at a density of 3 × 10^5^ cells/well and cultured for 0.5, 1, 2, and 4 h. The cell adhesive state was recorded using a microscope and quantified by two methods. Briefly, at each time point, adherent cells were digested with 0.25% ethylenediaminetetraacetic acid (EDTA)-trypsin (Gibco/Thermo Fisher Scientific Inc., Waltham, MA, USA) and counted using a Countess II automatic cell counter (Life Technologies/Invitrogen, Carlsbad, CA, USA). Alternatively, 400 μl of methyl thiazolyl tetrazolium (MTT) working solution (Sigma-Aldrich) was added at each time point. After incubation at 37°C for 4 h, the supernatant was discarded, and 2 mL of dimethyl sulfoxide (DMSO) (Sigma-Aldrich) was added to dissolve the blue formazan derivative. The optical density (OD) values were measured at a wavelength of 490 nm.

### 2.4. Scratch migration assay and Transwell chemotaxis assay

BMSCs were seeded in 6-well culture plates at a density of 5 × 10^5^ cells per well. 6 hours later, cells were adhered to the plates. The MSCM was replaced with serum-free Alpha-Minimal Essential Medium (α-MEM; Gibco/Thermo Fisher Scientific Inc.), and cells were cultured for an additional 24 h. Scratches perpendicular to the long axis of the culture plate were made in each well using a 1-mL pipette tip (Axygen Biosciences Inc., Union City, CA, USA). Cell migration was observed and photographed at 0, 6, 12, and 24 h after scratching using a microscope (Olympus Corporation). The relative width (relative width = area of the blank region/height) was measured at each time point using the Image-Pro Plus 6.0 software (Media Cybernetics, Rockville, MD, USA) and compared with that at 0 h to calculate the relative migration distance.

Transwell chambers with an 8-μm-pore membrane (Corning Inc.) were placed in 24-well plates. BMSCs (5 × 10^4^) were seeded into each chamber. Then, 100 µl of serum-free α-MEM (Gibco/Thermo Fisher Scientific Inc.) was added to the upper chamber, and 600 µl of α-MEM containing 15% fetal bovine serum (FBS; Gibco/Thermo Fisher Scientific Inc.) was added into the lower chamber. After 24 h of culture, cells that had crossed the membrane were fixed and stained with Giemsa staining solution (Solarbio, Beijing, China) for 30 min. Cells in random fields were counted under a microscope (Olympus Corporation).

### 2.5. Alkaline phosphatase (ALP) staining and activity assay

BMSCs were induced and cultured in an osteogenic-inducing medium according to the StemPro osteogenesis differentiation kit (Invitrogen) for 10 days. Then, the cells were fixed in 70% ethanol for 1 h and stained using an ALP staining kit (Beyotime, Shanghai, China) according to the manufacturer’s protocol. Intracellular ALP activity was measured at 3, 5, and 7 days of induction using an ALP activity assay kit (Nanjing Jiancheng Biotechnology Co. Ltd., Nanjing, China) according to the manufacturer’s protocol. The results were standardized relative to the protein concentration.

### 2.6. Alizarin red S staining

After 21 days of osteogenic induction, BMSCs were fixed in 70% ethanol and then stained for 5 min with a 2% alizarin red S staining solution (Sigma-Aldrich). Subsequently, 250 μl of isopropanol was added to each well to dissolve the red derivatives in the calcium nodules, and the OD was measured at a wavelength of 550 nm.   

### 2.7. Real-time reverse transcriptase-polymerase chain reaction (RT-PCR)

Total RNA was extracted from BMSCs using the TRIzol reagent (Invitrogen) and reverse transcribed into cDNA using the PrimeScript RT Reagent Kit and gDNA Eraser (Takara Bio Inc., Kusatsu, Shiga, Japan) according to the manufacturer’s protocol. Then, RT-PCR was performed using an SYBR Premix Ex Taq II reagent kit (Takara Bio Inc.) on an iCycler iQ™ Multi-color Real-time PCR Detection System (Bio-Rad Laboratories Inc., Hercules, CA, USA). Glyceraldehyde-3-phosphate dehydrogenase (*GAPDH*) was used as an internal control, and the 2^−ΔΔCt^ method was used for analysis. The primer sequences are listed in Table 1.

**Table 1.** Sequences of the primers used for real-time RT-PCR

Gene name	Primer Sequence (5’ to 3’)
*ITGA10*	Forward: AACATCACCCACGCCTATTCC
	Reverse: GTTGGTAGTCACCTAAGTGGC
*COL1A1*	Forward: GAGGGCCAAGACGAAGACATC
	Reverse: CAGATCACGTCATCGCACAAC
*OCN*	Forward: CACTCCTCGCCCTATTGGC
	Reverse: CCCTCCTGCTTGGACACAAAG
*BSP*	Forward: CAGGCCACGATATTATCTTTACA
	Reverse: CTCCTCTTCTTCCTCCTCCTC
*GAPDH*	Forward: ATGTGGATCAGCAAGCAGGA
	Reverse: GGTGTAAAACGCAGCTCAGTAA

### 2.8. Bioinformatics analysis of the whole-cell proteomics data

Based on the whole-cell proteomics datasets of BMSCs from the DM-F group and DM-S group (five samples in each group), which were deposited in ProteomeXchange with the identifier PXD016489 [15], the differentially expressed proteins with a fold change greater than 1.3 were extracted for subsequent analysis to avoid missing important protein information. Gene Ontology (GO) annotation of 247 differentially expressed proteins was performed using the FunRich software v2.1.2 (http://www.funrich.org/) for three terms, namely biological process, cellular component, and molecular function. The Kyoto Encyclopedia of Genes and Genomes (KEGG) pathway enrichment analysis was performed using the DAVID online tool v6.8 (https://david.ncifcrf.gov/), and a bubble chart was plotted using the R software v3.6.1 (https://cran.r-project.org/). Pathway maps were generated using the online KEGG tool (https://www.kegg.jp/). Interactions among differentially expressed proteins were analyzed using the online STRING tool v11.0 (https://string-db.org/), and a network of protein-protein interactions was generated using the Cytoscape software v3.7.1 (https://cytoscape.org/). The R software v3.6.1 was used to perform a random forest analysis [25,26] of the differentially expressed proteins and to generate multidimensional scaling plots. Differentially expressed proteins were sequenced based on the Mean Decrease Gini (MDG) coefficient, thereby providing a basis for extracting important proteins.

### 2.9. ITGA10 *knockdown and overexpression by lentiviral transfection*

Short hairpin RNAs (shRNA) targeting *ITGA10* complementary sequences (*ITGA10*-sh1: 5’-ACACACAAACAGACTGAAT-3’, *ITGA10*-sh2: 5’-GCTAAAGGATGGGATTCTT-3’) were inserted into the vector GV248 (hU6-MCS-Ubiquitin-EGFP-IRES-puromycin) to construct an *ITGA10*-knockdown plasmid, and full-length human *ITGA10* cDNA was inserted into the vector GV492 (Ubi-MCS-3FLAG-CBh-gcGFP-IRES-puromycin) to construct an *ITGA10*-overexpression plasmid. Lentiviral packaging was completed by GeneChem Co., Ltd. (Shanghai, China). The *ITGA10*-knockdown lentivirus, knockdown control lentivirus (GV248), *ITGA10*-overexpression lentivirus, and overexpression control lentivirus (GV492) were transfected into alveolar BMSCs from T2DM patients (DM-S group) with 5 μg/mL polybrene (Sigma-Aldrich) for 10 h. After 48 h, 10 mg/mL puromycin (Thermo Fisher Scientific Inc.) was used for screening transfected cells for 3 days to obtain stable *ITGA10*-knockdown and *ITGA10*-overexpression cell models. In addition, in the overexpression experiment, the focal adhesion kinase (FAK) inhibitor, PF573228 (Selleck Chemicals LLC, Houston, TX, USA), was added to the osteogenic-inducing medium at a final concentration of 10 μM to inhibit FAK phosphorylation.

### 2.10. Western blot analysis

Cells were lysed in RIPA buffer supplemented with 1% phenylmethylsulfonyl fluoride and 1% protease inhibitor cocktail (Sigma-Aldrich). Protein concentrations were determined using a bicinchoninic acid (BCA) Protein Quantitation Kit (Beyotime, Shanghai, China). The protein samples were separated by electrophoresis on a 15% sodium dodecyl sulfate polyacrylamide gel (Bio-Rad Laboratories Inc.) and transferred to polyvinylidene fluoride membranes (Bio-Rad Laboratories Inc.) using a semidry transfer apparatus (Bio-Rad Laboratories Inc.). Then, the membranes were blocked with 5% nonfat milk for 1 h. The corresponding primary antibody, diluted to the recommended concentration in accordance with the instruction manual, was then added, and the membranes were incubated at 4°C overnight. Subsequently, a horseradish peroxidase-conjugated goat anti-rabbit secondary antibody (1:5,000; ABclonal) was added, followed by incubation for 1 h. The membranes were then immersed in electrochemiluminescence solution (Bio-Rad Laboratories Inc.) and visualized using the ChemiDoc MP Imaging System (Bio-Rad Laboratories Inc.). The primary antibodies used included rabbit polyclonal anti-ITGA10 (Merck Millipore, Amsterdam, the Netherlands), rabbit monoclonal anti-p-FAK, anti-FAK, anti-p-PI3K, and anti-PI3K (Abcam), rabbit monoclonal anti-p-AKT, anti-AKT, anti-p-GSK3β, anti-GSK3β, and anti-β-catenin (Cell Signaling Technology, Danvers, MA, USA), and rabbit monoclonal anti-β-actin (ABclonal).

### 2.11. Statistical analysis

The SPSS software version 23.0 (IBM Corporation, Armonk, NY, USA) was used for statistical analysis. Statistical significance was determined by the Student’s t test or one-way analysis of variance (ANOVA). The level of significance was defined using two p-values (*: *P* < 0.05, **: *P* < 0.01).

## 3. Results

T2DM is considered an important risk factor for oral implant treatment failure. This study aimed to determine the molecular causes of the high implant failure rate in T2DM patients during the healing stage. We found that the adhesion, migration, and osteogenic differentiation abilities of alveolar BMSCs from diabetic patients with implant failure were significantly lower than those of diabetic patients with implant success and non-diabetic patients. Simultaneously, the expression of ITGA10 in alveolar BMSCs from diabetic patients with implant failure was also significantly lower than that in the other two groups. Furthermore, *ITGA10* knockdown significantly reduced BMSC adhesion and migration abilities and inhibited the osteogenic differentiation potential by disturbing the FAK/PI3K/AKT/GSK3β/β-catenin pathway, while *ITGA10* overexpression yielded the opposite results. The results revealed that the low ITGA10 expression in alveolar BMSCs from T2DM patients leads to cell dysfunction, which could be an important cause of early implant failure by lack of osseointegration.

### 3.1. Biological functions of BMSCs from the DM-F, DM-S, and Nor groups

**Figure 1.**
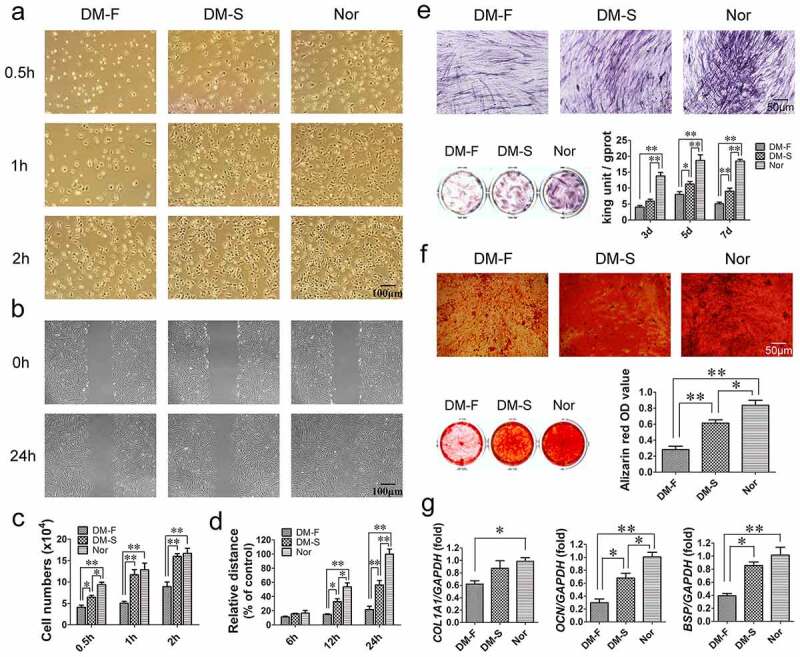
Comparison of biological functions of BMSCs from the DM-F, DM-S, and Nor groups. (a, c) The adhesion ability of BMSCs was significantly lower in the DM-F group than in the DM-S and Nor groups. (b, d) The migration ability of BMSCs in the DM-F group was lower than that in the DM-S group and much lower than that in the Nor group. (e) The results of the ALP staining and activity assay showed that the osteogenic differentiation ability of BMSCs in the DM-F group was lower than that in the DM-S group and much lower than that in the Nor group. (f) Alizarin red staining and quantitative results showed a trend similar to that of the ALP results. (g) The mRNA expression levels of *COL1A1, OCN*, and *BSP* in BMSCs were also significantly lower in the DM-F group. The results are expressed as the mean ± standard deviation. *P < 0.05; **P < 0.01.

Cell adhesion assays revealed that the adhesion states of BMSCs in the DM-F, DM-S, and Nor groups started to differ at 0.5 h, and at 1 and 2 h, the number of adherent BMSCs in the DM-F group was significantly lower than those in the other two groups (Figures 1a, c). The scratch migration assay showed that the migration ability of BMSCs in the DM-F group was lower than that in the DM-S group and much lower than that in the Nor group (Figure 1b). The quantitative results showed that at 12 and 24 h, the relative migration distance of BMSCs from the DM-F group was significantly shorter than that of BMSCs from the other two groups, while the distance in the DM-S group was significantly shorter than that in the Nor group (Figure 1d).

ALP and alizarin red staining were used to compare the osteogenic differentiation potential of the BMSCs in the three groups. The results revealed that the number of ALP-positive cells and the degree of staining in the DM-F group were significantly lower than those in the DM-S group and the Nor group had the most intense staining. The ALP activity of cells in the DM-F group and DM-S group were significantly lower than that in the Nor group on day 3, and from day 5, the ALP activity was significantly different among the three groups (Figure 1e). Alizarin red staining showed that the mineralization ability of BMSCs was lower in the DM-F group than in the DM-S group and lower in the DM-S group than in the Nor group (Figure 1f). Moreover, the osteogenesis-related mRNA expression results after 7 days of osteogenic induction showed that the *COL1A1* expression level in BMSCs from the DM-F group was lower than that in BMSCs from the Nor group, and the *OCN* and *BSP* expression levels in BMSCs from the DM-F group were significantly lower than those in BMSCs from the DM-S and Nor groups (Figure 1g).

### 3.2. Abnormally expressed proteins and abnormal functions of BMSCs from diabetic patients with implant failure

**Figure 2.**
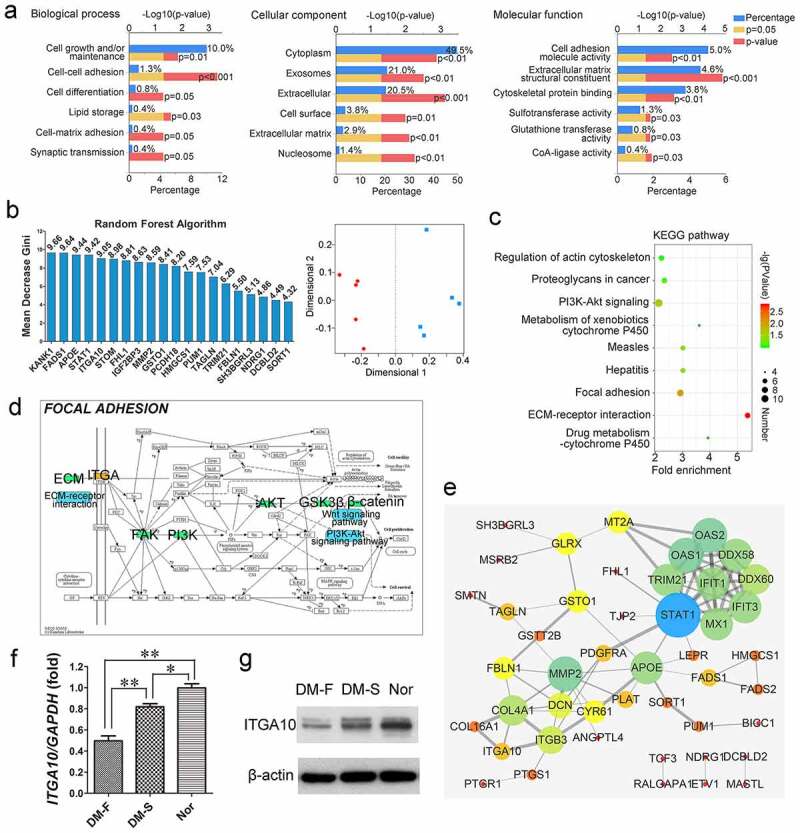
Proteomics-based bioinformatics analysis results. (a) GO annotation showed the biological processes, cellular components, and molecular functions of differentially expressed proteins in BMSCs from the DM-F and DM-S groups. (b) Random forest analysis showed that the Mean Decrease Gini coefficient of ITGA10 was 9.05, ranking fifth among all differentially expressed proteins, and the multidimensional scaling plots showed good intragroup similarity in the DM-F group and the DM-S group. (c) KEGG analysis showed significant differences in integrin function-related signaling pathways between the above two groups. (d) Diagram of the focal adhesion signaling pathway showed the relations of ECM/ITG/FAK/PI3K/AKT/GSK3β/β-catenin. (e) Protein-protein interaction analysis of differentially expressed proteins showed strong correlations between ITGA10 and COL16A1, ITGA10 and COL4A1, and ITGA10 and ITGB3. (f, g) Real-time RT-PCR and western blot results confirmed that ITGA10 expression was significantly lower in the DM-F group than in the DM-S group, and ITGA10 expression in both of these groups was lower than that in the Nor group. The results are expressed as the mean ± standard deviation. *P < 0.05; **P < 0.01.

GO analysis of differentially expressed proteins in BMSCs from the DM-F and DM-S groups showed that for the biological process category, ‘cell growth and/or maintenance’-related proteins accounted for the highest proportion of differentially expressed proteins, and the ‘cell-cell adhesion’ process exhibited the most statistically significant difference. For the cellular component category, differentially expressed proteins were mainly found in ‘cytoplasm’, followed by ‘exosomes’ and ‘extracellular’. For the molecular function category, the main functions of the differentially expressed proteins were ‘cell adhesion molecule activity’ and ‘extracellular matrix structural constituent’ (Figure 2a). The top 10 protein rankings in terms of the fold change in differential expression (*P* < 0.05) are listed in Table 2, which reveals that ITGA10 expression in BMSCs from the DM-F group was 0.655 times that in BMSCs from the DM-S group, with a *P* value of 0.00108. The random forest analysis results showed that the MDG coefficient of ITGA10 was 9.05, ranking fifth among all 247 differentially expressed proteins (the top 20 are shown in Figure 2b). Moreover, multidimensional scaling plots showed that the protein expression data from the DM-F and DM-S groups had an acceptable intragroup similarity, indicating that the random forest results were stable and reliable (Figure 2b).

**Table 2.** Top 10 significantly differentially expressed proteins in the DM-F group/DM-S group

Upregulated			Downregulated		
Protein name	Fold change	*P* value	Protein name	Fold change	*P* value
IFIT1	3.243	0.000802	APOE	0.478	0.000451
IFIT3	2.417	0.00642	TMEM119	0.565	0.0157
MX1	2.411	0.0223	MMP2	0.571	0.00868
ERAP2	1.775	0.0194	FBLN1	0.579	0.00593
OAS2	1.692	0.0166	OR10G3	0.605	0.0107
TAGLN	1.619	0.00779	CRISPLD2	0.611	0.0119
MSRB2	1.614	0.0244	PHF12	0.633	0.049
DCBLD2	1.496	0.0146	CENPV	0.638	0.0158
KANK1	1.45	0.000913	ITGA10	0.655	0.00108
ALDH1B1	1.446	0.0427	COL16A1	0.672	0.0329

The KEGG pathway enrichment analysis results are shown in Figure 2c. Among the enriched KEGG pathways associated with the differentially expressed proteins, the ‘Focal adhesion’, ‘PI3K-Akt signaling’, ‘Regulation of actin cytoskeleton’, and ‘ECM-receptor interaction’ KEGG pathways are closely related to integrin functions. The focal adhesion pathway diagram showed that integrin binding by ECM proteins can promote FAK phosphorylation, activate the PI3K-AKT signaling pathway, further phosphorylate GSK3β to inhibit its activity, reduce the degradation of β-catenin, and promote the translocation of β-catenin into the nucleus to regulate biological processes in cells (Figure 2d). In addition, analysis of protein-protein interaction of the differentially expressed proteins showed close correlations of ITGA10 with COL16A1, COL4A1, and ITGB3 (Figure 2e). The expression level of COL16A1 in BMSCs from the DM-F group was 0.672 times that in BMSCs from the DM-S group, and the *P* value was 0.0329 (Table 2). The differential trend for COL16A1 was similar to that for ITGA10.

Real-time RT-PCR and Western blot analyses were used to verify the expression level of ITGA10. The results showed that the *ITGA10* mRNA expression level in the DM-F group was significantly lower than that in the other two groups and that in the DM-S group was lower than that in the Nor group (Figure 2f). A similar trend to that of the RT-PCR analysis was shown by the Western blot analysis results (Figure 2g).

### 3.3. ITGA10 *knockdown inhibits the adhesion and migration abilities of BMSCs from T2DM patients*

**Figure 3.**
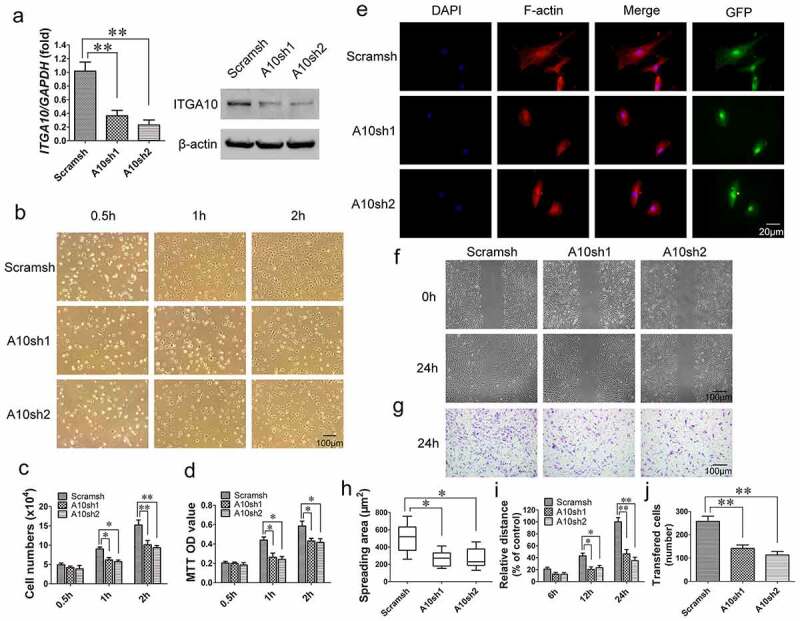
The inhibitory effect of ITGA10 knockdown on the adhesion and migration abilities of BMSCs. (a) Real-time RT-PCR and western blotting confirmed the knockdown efficiency. (b-d) ITGA10 knockdown significantly decreased the adhesion ability of BMSCs from T2DM patients. (e, h) ITGA10 knockdown significantly reduced the spreading area of BMSCs from T2DM patients. **(f, g, i, j)** Scratch and transwell assays showed that ITGA10 knockdown significantly reduced the migration ability of BMSCs from T2DM patients. The results are expressed as the mean ± standard deviation. *P < 0.05; **P < 0.01.

*ITGA10* was knocked down in BMSCs from T2DM patients using two different *ITGA10* shRNA lentiviruses (A10sh1 and A10sh2). After puromycin selection of the transfected cells, real-time RT-PCR and Western blot analyses confirmed the efficient knockdown of *ITGA10* expression in BMSCs (Figure 3a).

Cell adhesion assays showed that *ITGA10* knockdown inhibited the adhesion ability of alveolar BMSCs from T2DM patients (Figure 3b). Cell counting and MTT assay quantitative results showed that starting from 1 h, the number of adherent cells and the OD value in the *ITGA10*-knockdown group were significantly lower than those in the control group (Figures 3c, d). The results of the F-actin immunofluorescence staining showed that the spreading area of BMSCs in the *ITGA10*-knockdown groups was significantly lower than that in the control group (Figures 3e, h).

The results of the scratch migration and Transwell assays indicated that *ITGA10* knockdown inhibited the migration ability of BMSCs from T2DM patients (Figures 3f, g). The relative migration distance of BMSCs from the *ITGA10*-knockdown groups was shorter than that of BMSCs from the control group at 12 h, and the difference was more significant at 24 h (Figure 3i). The transferred cell counting results also showed that the number of migrated cells in the *ITGA10*-knockdown groups was significantly lower than that in the control group (Figure 3j).

### 3.4. ITGA10 *knockdown inhibits the osteogenic differentiation potential of BMSCs from T2DM patients*

**Figure 4.**
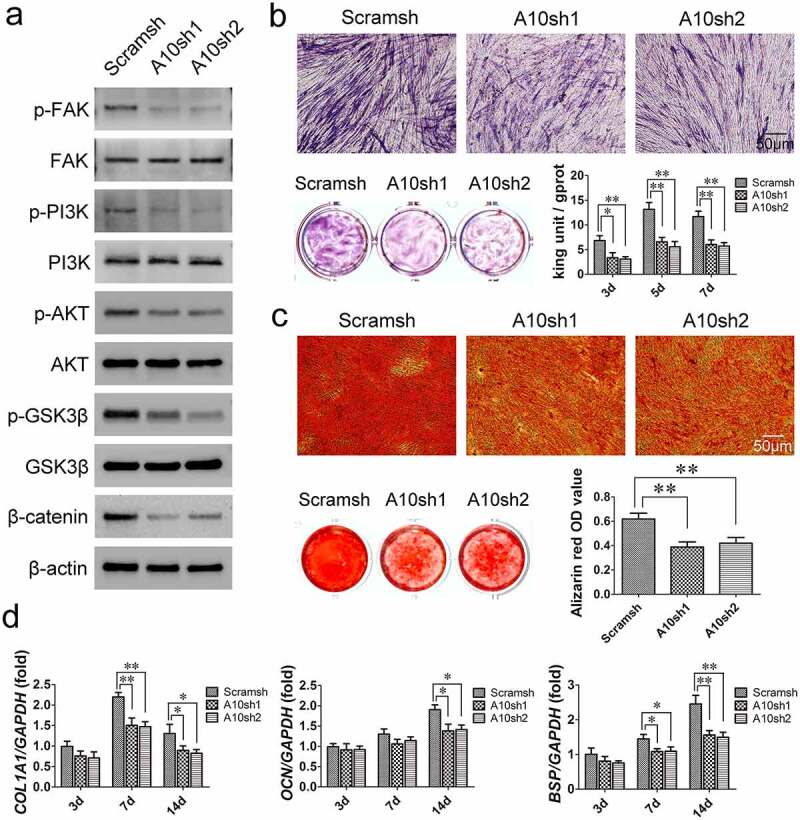
The inhibitory effect of ITGA10 knockdown on the osteogenic differentiation potential of BMSCs. (a) Western blot results showed that ITGA10 knockdown downregulated the FAK/PI3K/AKT/GSK3β/β-catenin signaling pathway. (b) After ITGA10 knockdown, BMSCs from T2DM patients exhibited significantly decreased ALP expression and activity. (c) After ITGA10 knockdown, the mineralization ability of BMSCs from T2DM patients was also significantly decreased. (d) ITGA10 knockdown resulted in significant decreases in *COL1A1* and *BSP* mRNA expression in BMSCs from T2DM patients after 7 days of osteogenic induction; after 14 days, the mRNA expression levels of *COL1A1, OCN*, and *BSP* were all significantly decreased. The results are expressed as the mean ± standard deviation. *P < 0.05; **P < 0.01.

According to the KEGG pathway enrichment analysis results (Figures 2c, d), Western blot analysis was used to detect changes in the FAK/PI3K/AKT/GSK3β/β-catenin signaling pathway in BMSCs from T2DM patients after 7 days of osteogenic induction. The results revealed that *ITGA10* knockdown reduced the phosphorylation level of FAK at Y397, and as a result, PI3K/AKT signaling was inhibited, the phosphorylation level of GSK3β was reduced, and β-catenin dissociated (Figure 4a). The results of ALP staining and activity assays showed that after *ITGA10* knockdown, the ALP expression in BMSCs from T2DM patients was significantly decreased (Figure 4b). Similarly, the alizarin red staining results showed that the mineralization ability of the BMSCs was also significantly decreased (Figure 4c). Furthermore, after 7 days of osteogenic induction, *ITGA10* knockdown resulted in a significant decrease in *COL1A1* and *BSP* expression in BMSCs, and after 14 days, the mRNA expression levels of *COL1A1, OCN*, and *BSP* were all significantly lower in the two knockdown groups (Figure 4d).

### 3.5. ITGA10 *overexpression enhances the adhesion and migration abilities of BMSCs from T2DM patients*

**Figure 5.**
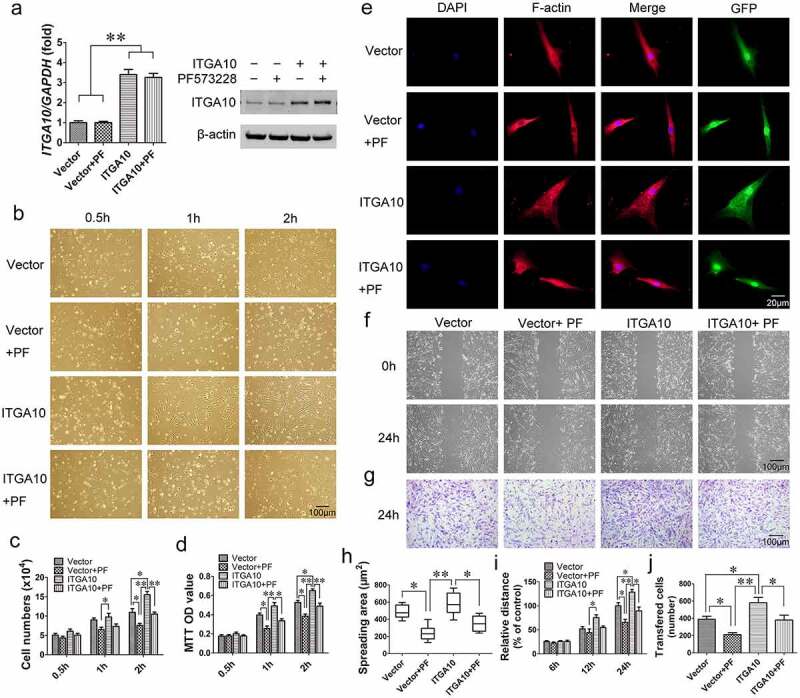
The enhancement effect of ITGA10 overexpression on the adhesion and migration abilities of BMSCs. (a) Real-time RT-PCR and western blotting confirmed the overexpression efficiency. (b-d) ITGA10 overexpression significantly increased the adhesion ability of BMSCs from T2DM patients, whereas the FAK inhibitor PF573228 significantly inhibited this effect. (e, h) ITGA10 overexpression led to a slight increase in the spreading area of BMSCs from T2DM patients, whereas PF573228 significantly reduced the spreading ability of these BMSCs. **(f, g, i, j)** Scratch and transwell assays showed that ITGA10 overexpression significantly increased the migration ability of BMSCs from T2DM patients, but PF573228 significantly reduced this effect. The results are expressed as the mean ± standard deviation. PF represents PF573228. *P < 0.05; **P < 0.01.

Real-time RT-PCR and Western blot analyses were performed to determine the ITGA10 mRNA and protein expression levels in BMSCs from T2DM patients in the control vector group (Vector group), control vector + PF573228 group (Vector + PF group), *ITGA10*-overexpression group (ITGA10 group), and *ITGA10*-overexpression + PF573228 group (ITGA10 + PF group). The results confirmed the overexpression efficiency and that the FAK inhibitor PF573228 did not affect *ITGA10* overexpression (Figure 5a).

Cell adhesion assays revealed that *ITGA10* overexpression enhanced the adhesion ability of BMSCs from T2DM patients, while PF573228 significantly reduced the enhancement effect (Figure 5b). The cell counting and MTT assay quantitative results were consistent with the microscopy results (Figures 5c, d). F-actin immunofluorescence staining and the spreading area calculation indicated that *ITGA10* overexpression led to a slight increase in the spreading ability of BMSCs, but the difference was not statistically significant, although PF573228 significantly reduced the spreading area (Figures 5e, h).

The results of the scratch migration assay and Transwell assay showed that *ITGA10* overexpression enhanced the migration ability of BMSCs from T2DM patients. After the addition of PF573228, the migration ability of the BMSCs was significantly reduced in both the ITGA10 + PF group and the Vector + PF group (Figures 5f, g, i, j).

### 3.6. *ITGA10* overexpression enhances the osteogenic differentiation potential of BMSCs from T2DM patients through the FAK/PI3K/AKT/GSK3β/β-catenin signaling pathway

**Figure 6.**
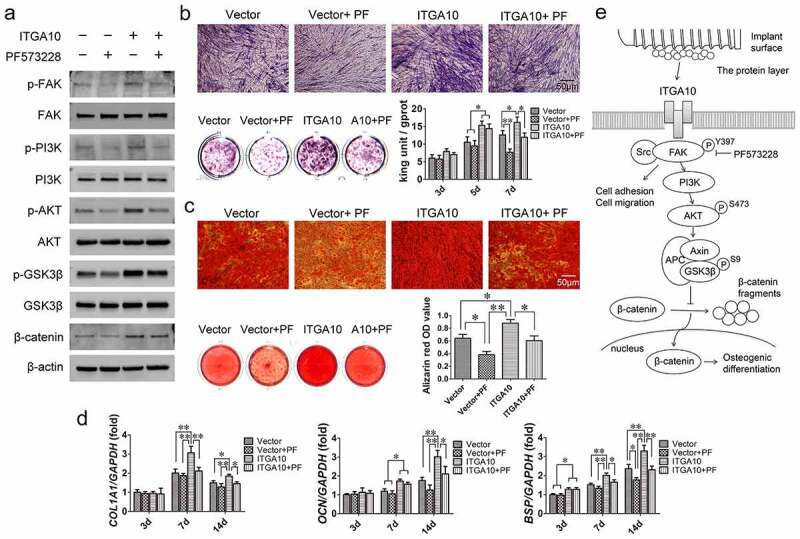
ITGA10 overexpression enhanced the osteogenic differentiation potential of BMSCs through the FAK/PI3K/AKT/GSK3β/β-catenin signaling pathway. (a) Western blot results showed that ITGA10 overexpression activated the FAK/PI3K/AKT/GSK3β/β-catenin signaling pathway and that PF573228 effectively inhibited this pathway. (b) ITGA10 overexpression resulted in a significant increase in ALP expression and activity in BMSCs from T2DM patients. (c) ITGA10 overexpression also significantly increased the mineralization ability of BMSCs from T2DM patients. (d) The mRNA expression levels of *COL1A1, OCN*, and *BSP* were significantly increased after ITGA10 overexpression. PF573228 significantly inhibited the enhancement effect of ITGA10 overexpression on osteogenic differentiation of BMSCs from T2DM patients. (e) ITGA10 regulated the osteogenic differentiation potential of BMSCs via the FAK/PI3K/AKT/GSK3β/β-catenin signaling pathway. The results are expressed as the mean ± standard deviation. PF represents PF573228. *P < 0.05; **P < 0.01.

The Western blot analysis results showed that *ITGA10* overexpression promoted the phosphorylation levels of FAK/PI3K/AKT/GSK3β and increased the expression of β-catenin after 7 days of osteogenic induction, while PF573228 significantly decreased the activation of this signaling pathway (Figure 6a). The ALP assay showed that *ITGA10* overexpression increased ALP expression and intracellular ALP activity in BMSCs from T2DM patients, while PF573228 interfered with the promotion effect (Figure 6b). The trend of the alizarin red staining and quantitative results were similar to the ALP assay results (Figure 6c). Moreover, the results of the measurement of the mRNA expression of osteogenesis-related genes showed that *ITGA10* overexpression led to a significant increase in *COL1A1, OCN*, and *BSP* mRNA expression in BMSCs from T2DM patients, while PF573228 significantly inhibited the upregulatory effect (Figure 6d). These results confirmed that *ITGA10* overexpression enhanced the osteogenic differentiation potential of BMSCs from T2DM patients through the FAK/PI3K/AKT/GSK3β/β-catenin signaling pathway (Figure 6e).

## 4. Discussion

T2DM has long been considered a relative contraindication to dental implant surgery [2,27]. However, due to the complex pathology of diabetes mellitus, the causes and mechanism of the high risk of dental implantation failure in patients with T2DM are still unclear. *In vivo* studies have shown that the formation of new bone around implants was not stable in hyperglycemic Goto-Kakizaki (GK) rats [28], and during the process of implant osseointegration, the proliferation and osteogenic differentiation of the surrounding BMSCs was clearly abnormal [29]. Cell studies have also revealed that a high-glucose environment can accelerate the aging of BMSCs and reduce their biological activity [30]. However, T2DM is a complex syndrome caused by islet dysfunction, and hyperglycemia is only one of its clinical features [31]. Normal BMSCs cultured in a high-glucose environment cannot fully reflect the status of BMSCs from T2DM patients. In addition, BMSCs from GK rats also cannot represent the condition of human alveolar BMSCs in diabetic patients.

Therefore, BMSCs isolated directly from the alveolar bone of T2DM patients are a relatively ideal cell model for *in vitro* studies. This study showed that the adhesion, migration, and osteogenic differentiation abilities of alveolar BMSCs from T2DM patients were significantly lower than those of alveolar BMSCs from nondiabetic patients, and the above abilities were significantly diminished in BMSCs from diabetic patients with implant failure compared with those from diabetic patients with implant success. These results indicated that the biological functions of alveolar BMSCs were closely related to the success or failure of implant osseointegration in the three groups of clinical patients evaluated in this study. When the biological functions of alveolar BMSCs in T2DM patients were reduced to a certain extent, abnormal osseointegration could be triggered, and early implant failure could occur.

Previous studies have shown that the bone-derived cells have to adhere first to the implant surface for the cells to perform subsequent functions [32]. After an implant is inserted into the alveolar bone, specific ECM proteins in the blood rapidly adsorb onto the surface of the implant to form a ‘protein layer’ [33], and BMSCs recognize the Arg-Gly-Asp (RGD) tripeptide sequence of proteins in the protein layer and accumulate at the implant-bone interface [34]. BMSCs then anchor on the implant surface under the action of integrins and focal adhesions to initiate the subsequent cell spreading, migration and osteogenic differentiation functions [35]. Eventually, the implant can gradually achieve stable integration with the alveolar bone of the patient during the healing period. Therefore, a good adhesion performance of alveolar BMSCs is beneficial for the subsequent osteogenic differentiation process and promotes early osseointegration [36,37]. However, abnormal BMSC adhesion will result in a reduced differentiative ability and induce apoptosis, which is an important cause of osseointegration failure [38]. In this study, both bioinformatics analysis and cell adhesion assays indicated that the adhesion ability of BMSCs in the DM-F group was significantly lower than that in the DM-S group, further implying that the abnormal adhesion function of BMSCs indeed is among the important factors leading to clinical implant osseointegration failure in the DM-F group patients.

The protein group on which cell adhesion depends includes mainly ECM proteins, transmembrane proteins, and cytoskeleton proteins [39]. Among these proteins, integrins, which are an important class of transmembrane proteins, can not only influence cell adhesion, but also regulate the differentiation ability of stem cells by activating downstream signaling pathways [40–42]. This study found that ITGA10 expression was significantly lower in BMSCs from diabetic patients with implant failure than that in BMSCs from diabetic patients with implant success and normal patients without diabetes. However, studies on the biological function and mechanism of ITGA10 in BMSCs are currently lacking. In this study, lentiviral transfection experiments confirmed that *ITGA10* knockdown in BMSCs from diabetic patients significantly inhibited cell adhesion, migration, and osteogenic differentiation abilities, which were enhanced when *ITGA10* was overexpressed. Therefore, low ITGA10 expression in BMSCs in diabetic patients with implant failure could affect the biological functions of the cells, which might be an important cause of abnormal implant osseointegration in these patients during the healing phase.

Integrins can signal BMSCs to activate the cytoskeleton to form an interconnecting network structure near the cell membrane, namely focal adhesion complexes, and after focal adhesion assembly, the cells begin to spread to maintain their normal phenotype and subsequent functions [43]. The spreading of BMSCs is another indispensable process for the success of osseointegration during the early healing phase [44]. In this study, immunofluorescence staining after *ITGA10* knockdown and overexpression revealed that the expression level of ITGA10 was positively correlated with the cell spreading ability. Therefore, ITGA10 could have an effect by regulating focal adhesion assembly, which is clinically important for the early osseointegration of the implant.

FAK is the most important component of the focal adhesion signaling pathway. After the adhesion of bone-derived cells to the surface of biological materials, chemical signals from the ECM and the surface of the biological materials are recognized by integrins and transduced into the cells to activate the downstream FAK and related signaling networks for further regulation of the differentiation process of the cells [45–47]. In this study, *ITGA10* knockdown led to a significant decrease in FAK phosphorylation levels in BMSCs from T2DM patients. Previous studies have shown that reduced FAK phosphorylation significantly affects the expression of osteogenesis-related proteins in mesenchymal stem cells, such as ALP, as well as the degree of matrix mineralization [48], and these findings were confirmed in the present study. Additionally, the results of this study showed that FAK phosphorylation in alveolar BMSCs from T2DM patients effectively activated the downstream PI3K/AKT signaling pathway. Earlier studies have demonstrated that the PI3K/AKT signaling pathway is involved in multiple biological processes in BMSCs, mainly including cell proliferation and osteogenic differentiation [49]. PI3K/AKT and its downstream target protein GSK3β are considered to be key factors in bone formation and reconstruction 50,51. Our study found that *ITGA10* knockdown significantly downregulated the above-mentioned signaling pathway, reduced the mineralization ability of the cells, and inhibited the expression of osteogenesis-related genes (*COL1A1, OCN*, and *BSP*). Therefore, the negative regulation of the focal adhesion signaling pathway through low ITGA10 expression can adversely affect the osteogenic function of alveolar BMSCs in T2DM patients and thus disturb implant osseointegration during the healing phase.

The canonical Wnt/β-catenin signaling pathway is an important pathway that regulates osteoblast differentiation and new bone formation. Several studies have shown that AKT activation can induce the phosphorylation of downstream GSK3β, prevent the degradation of β-catenin by the APC-axin-GSK3β complex and promote its aggregation, thereby activating the Wnt/β-catenin signaling pathway, stimulating the expression of osteogenic factors, and promoting the osteogenic differentiation of mesenchymal stem cells [52,53]. In this study, *ITGA10* overexpression effectively activated the β-catenin signaling and increased the expression of osteogenesis-related genes *via* the above pathway. Based on the comprehensive analysis of the results, the upregulation of ITGA10 expression in BMSCs from T2DM patients can not only improve cell adhesion and migration abilities but also increase the osteogenic differentiation potential of BMSCs by regulating the FAK/PI3K/AKT/GSK3β/β-catenin signaling pathway. ITGA10 could be an important factor in the regulation of biological processes in BMSCs and a new marker for the evaluation of implant osseointegration in T2DM patients. In future studies, we will consider ITGA10 an intervention target and focus on how to regulate ITGA10 expression effectively and conveniently in alveolar BMSCs so as to improve cell function and implant osseointegration in T2DM patients and broaden indications for dental implant treatment.

## 5. Conclusions

This study revealed that the adhesion and migration abilities and osteogenic differentiation potential of alveolar BMSCs in diabetic patients with implant failure were significantly abnormal. The decreased expression of ITGA10 in BMSCs from diabetic patients with implant failure is an important cause of cell dysfunction. Furthermore, ITGA10 was found to be positively correlated with the adhesion and migration abilities of the BMSCs and could affect the osteogenic differentiation potential of BMSCs through the FAK/PI3K/AKT/GSK3β/β-catenin signaling pathway. The above findings reflect the potential of ITGA10 as an intervention target protein for improving the biological functions of BMSCs and clinical implant osseointegration in T2DM patients.

## Supplementary Material

Supplemental Material

## Data Availability

The whole-cell proteomics datasets for BMSCs from the DM-F, DM-S, and Nor groups are available via ProteomeXchange repository with the identifier PXD016489 (https://www.ebi.ac.uk/pride/archive/projects/PXD016489).
