# Supplementary figures and images for: Integrin α10 regulates adhesion, migration, and osteogenic differentiation of alveolar bone marrow mesenchymal stem cells in type 2 diabetic patients who underwent dental implant surgery
Source: Bioengineered. 2022 May 29;13(5):13252–68. doi: 10.1080/21655979.2022.2079254 (PMC9275886; doi:10.1080/21655979.2022.2079254)

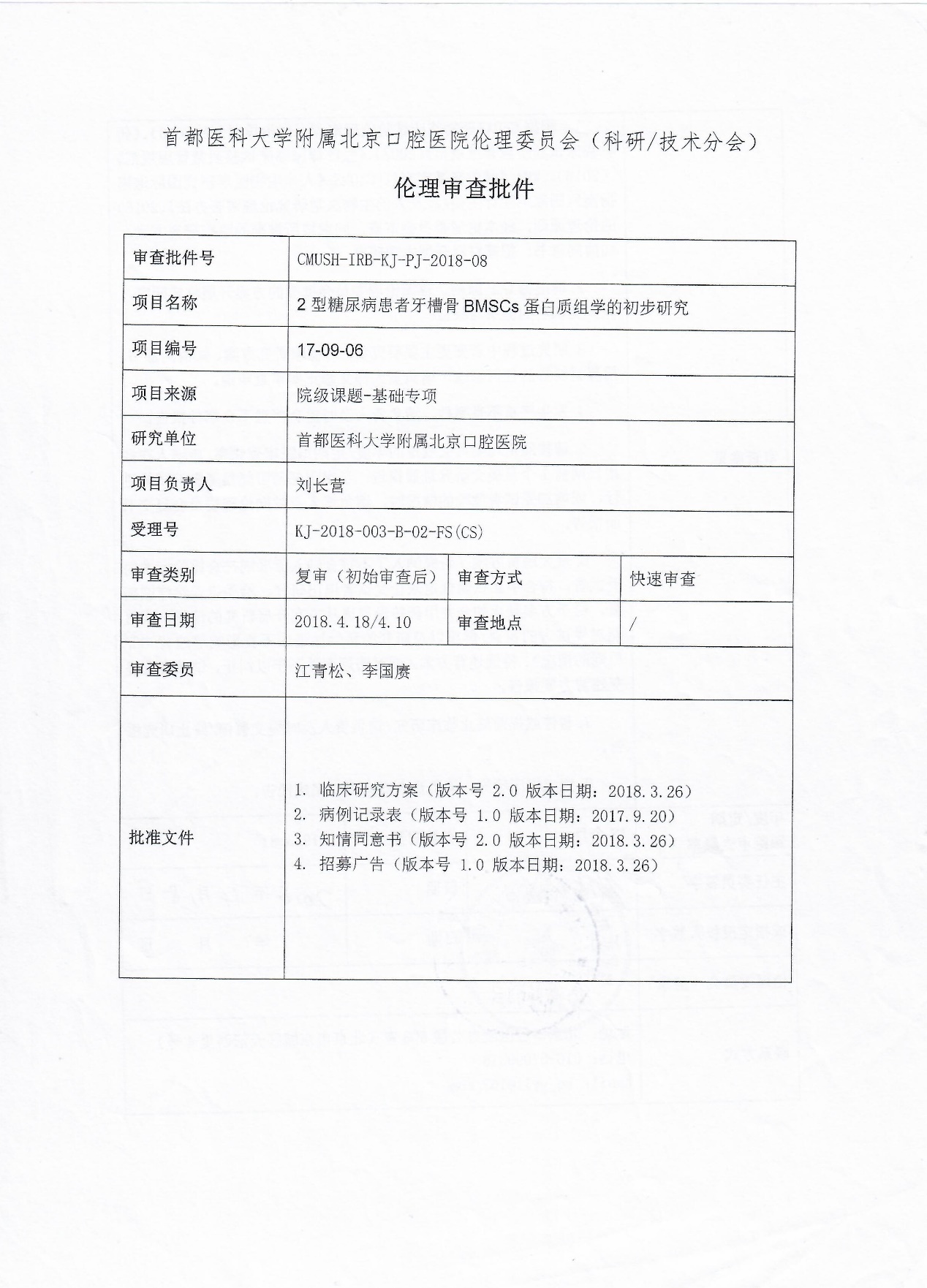


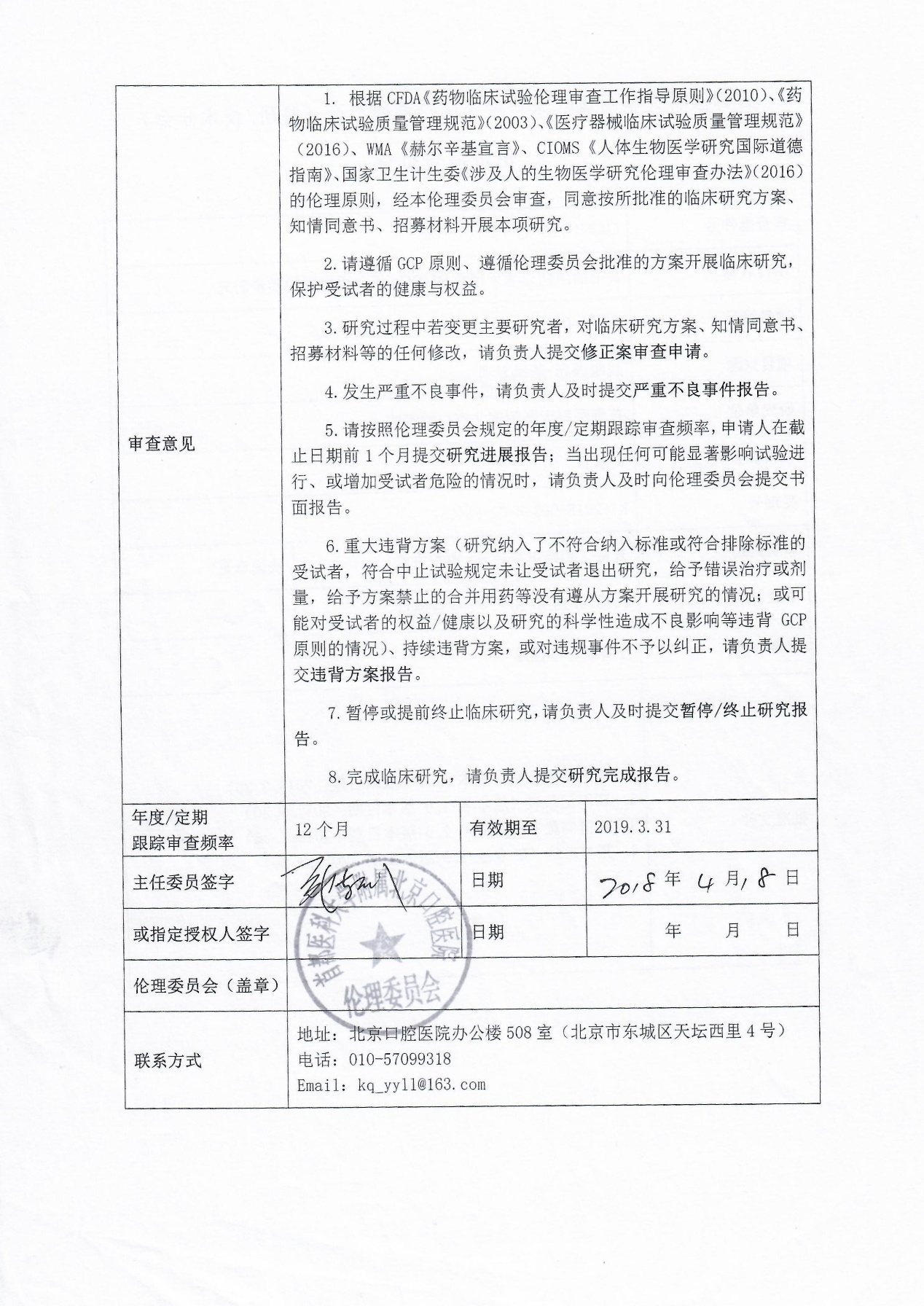

Supplement: Supplemental Material [file KBIE_A_2079254_SM8064.zip › supplementary/Ethical approvement.docx]
